# Neural changes in microgravity: Investigating the effects of aerospace environments

**DOI:** 10.1016/j.ibneur.2025.11.011

**Published:** 2025-11-15

**Authors:** Fateme Arjmand, Soroush Taherkhani, Aidin Shahrezaei, Maryam Sohani, Sanaz Sadeghi Esfahani, Sepideh Marjaei, Farinaz Nasirinezhad

**Affiliations:** a Reproductive Sciences and Technology Research Center, Department of Anatomical Sciences, Iran University of Medical Sciences, Tehran, Iran; b Department of Physiology, School of Medicine, Iran University of Medical Sciences, Tehran, Iran; c School of Medicine, Iran University of Medical Sciences, Tehran, Iran; d Department of Molecular Biotechnology, Cell Science Research Center, Royan Institute for Biotechnology, ACECR, Isfahan, Iran; e Department of Electrical, Computer, Information Technology and Biomedical Engineering, Qazvin Islamic Azad University, Qazvin, Iran; f Center of Experimental and Comparative Study, Iran University of Medical Sciences, Tehran, Iran

**Keywords:** Aerospace environments, Neural system, Brain activity, Microgravity

## Abstract

Technological, human, and financial advances have led to an increase in space exploration and, as a result, commercial space travel. Companies such as Space X and Blue Origin are exploring ways to make space travel possible. As a result, the number of patients with space-related neurological disorders may increase. Therefore, the management of neurological disorders in high-risk patients requires a thorough understanding of spaceflight exposure. The neural system physiology is significantly impacted by aerospace environments through various mechanisms including hypoxia, decompression sickness, cardiovascular system adaptations to microgravity and acceleration forces. These effects underscore the importance of understanding and mitigating these physiological changes to ensure the safety and well-being of individuals operating in aerospace environments. Potential benefits of spaceflight on the nervous system include psychological growth and improvements in memory and learning. Future space travelers who lack the physiological reserve of current astronauts may experience neurological problems as a result of the physiological and psychological stresses of space travel. As we continue to understand more about human adaptation to microgravity and the potential medical problems that may arise, the capabilities of healthcare must continue to progress to match the ambition of multinational corporations. In conclusion, there appear to be many factors that can affect the nervous system and lead to neurological diseases in people who travel to space. As space travel becomes more accessible to the general public these nervous system effects will need to become a staple of the future neurologist's clinical practice.

## Highlights

• Spaceflight induces short-term neurological changes, including impaired balance and cognition.
• Long-term risks include brain cancer, accelerated neurodegeneration, and early aging.
• Oxidative stress, triggered by microgravity, radiation, and isolation, is a key mechanism of neural damage.

## Graphical Abstract

**Figure:**
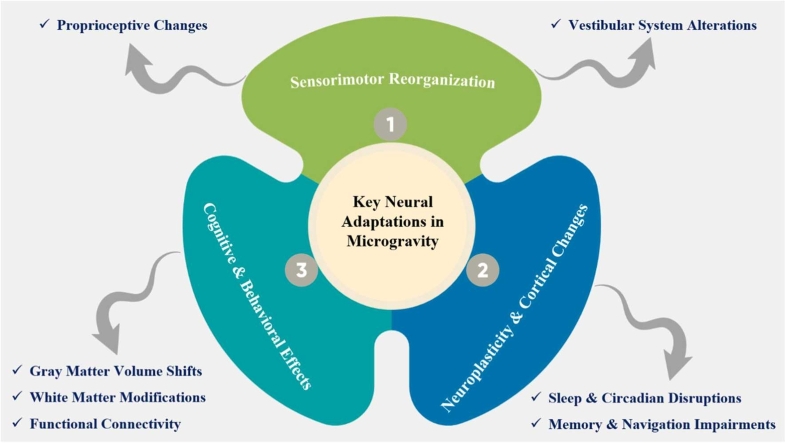
Microgravity induces significant physiological and neurological adaptations. The human nervous system, finely tuned to Earth’s gravity, undergoes structural and functional changes when exposed to prolonged microgravity. The main parts of this content are: (1) Sensorimotor Reorganization; (2) Neuroplasticity & Cortical Changes; (3) Cognitive & Behavioral.

## 1 Introduction

Space travel has become an increasingly vital aspect of modern exploration, with both government agencies and private companies investing heavily in sending humans to space. However, as we continue to push the boundaries of space exploration, it is essential to understand the effects of aerospace environments on the human body (Demontis et al., 2017). One of the most critical areas of concern is the impact of space travel on the neural system physiology. Prolonged exposure to microgravity, radiation, and other environmental stressors can have significant effects on the nervous system, leading to changes in cognitive function, motor control, and overall well-being (Van Ombergen et al., 2017a). The best-known stressor, microgravity, damages the vestibular system and the structural integrity of the eyes. Cosmic radiation has been linked to increased risk of cancer and neurological disorders. Psychological effects may result from these stressors in addition to confinement, isolation, and circadian rhythm disruption (Gupta et al., 2023).

Humans have long ventured into harsh environments-from the deep sea to outer space-in an attempt to find the biological elements necessary for existence. One of these harsh environments is space (Jemison and Olabisi, 2021). Although there are many environmental stressors that astronauts encounter in space, it is still unclear how much of an impact they have on the central nervous system (CNS) (Roy-O'Reilly et al., 2021). During extended space missions, astronauts are exposed to more stressors that will affect them both during and after their return to Earth. Microgravity, low gravity, prolonged hypercapnia, cosmic radiation, isolation, solitary confinement, and disrupted circadian rhythms are among the hazards astronauts face. These stresses can have long-term effects on the brain, lasting several years (Zwart et al., 2021).

The effects of aerospace environments on the neural system physiology are multifaceted and primarily revolve around the impact of altered atmospheric conditions and gravitational forces on the human body. At high altitudes, the decreased oxygen concentration and pressure can lead to hypoxia, causing disruptions in cortical functions and potentially leading to unconsciousness if not corrected. Additionally, decompression sickness can occur, where nitrogen dissolved in the blood and tissues forms bubbles upon rapid decompression, leading to potential tissue damage and organ failure (Moon, 2014) These effects can lead to significant physiological changes, particularly affecting the cardiovascular system, which in turn impacts the nervous system due to the interconnected nature of these systems (Bourassa et al., 2021, Tahsili-Fahadan and Geocadin, 2017).

In this review, we look at the CNS effects of spaceflight and suggest which nervous system disorders may preclude people from becoming space tourists.

## 2 Neurophysiological and structural brain responses to spaceflight

The neural system is a complex network of neurons, glial cells, and other supporting structures that work together to regulate various bodily functions, including movement, sensation, and cognitive processes (Li et al., 2025). In space, the neural system is exposed to unique environmental stressors that can disrupt its normal functioning. Microgravity, in particular, can cause changes in the way the brain processes information, leading to alterations in spatial awareness, balance, and movement (Van Ombergen et al., 2017b). Studies have shown that microgravity can cause changes in the way the brain processes visual information, leading to impaired balance and coordination. Additionally, microgravity can affect the functioning of the vestibular system, which is responsible for maintaining balance and spatial orientation (Kornilova et al., 2012).

Studies of astronauts following their trips to the International Space Station (ISS) have shown that spaceflight causes a number of changes in the human brain, some of which are likely to be more pronounced in deep space (Burles and Iaria, 2023). These include changes in the micro- and macrostructure of gray matter, adjustments in the distribution and composition of cerebrospinal fluid (CSF), and (Jillings et al., 2020). Exposure to real and simulated microgravity has caused anatomical and functional changes in the vestibular system's ability to control responses and actions at various stages of vestibular processing (Buoite Stella et al., 2021). The degradation of synaptic integrity and neuronal structure may account for some of the functional behavioral decline. In addition to increased radiation exposure, microgravity exposure will be a critical factor to consider. It is well known that the structural, functional and physiological functions of the human body are affected by microgravity (Willey et al., 2021). Prolonged spaceflight increases the total intracranial volume of the brain. This, along with increases in intracranial and intraocular pressure, causes structural changes in the brain (Marshall-Goebel et al., 2021). Increased intracranial pressure is likely the cause of conditions such as Spaceflight-Associated Neuro-Ocular Syndrome (SANS), formerly known as Visual Impairment and Intracranial Pressure (VIIP), which also causes neurovestibular problems and other performance-impairing effects such as cognitive impairment (Wusk et al., 2019).

Another significant hazard will be extended periods of isolation beyond what astronauts have experienced in low-Earth orbit. Astronauts will spend a significant amount of time in an isolated, confined, and intense (ICE) environment at the Lunar Gateway due to its distance from Earth compared to ISS missions (Arquilla et al., 2022). Both the short- and long-term effects are expected to be different from those of the International Space Station (ISS), based on previous human research conducted in ICE habitats, including space environment analogs (Kjærgaard et al., 2022). These include impaired cognitive function, immune response triggers from the environment, and the physiological and psychological effects of an intense environment. Extended periods of isolation can lead to various psychological stresses that negatively affect behavior and performance (Palinkas and Suedfeld, 2021). According to some research, sleeping in an ICE environment can have a detrimental effect on one's ability to function during a mission, leading to further stress and mental health issues. Isolation poses risks beyond its effects on the mind and body, as it has been shown to cause anatomical changes in the brain (Roalf et al., 2025).

## 3 Effects of microgravity on the neural system

Studies have shown that microgravity can affect the human neural system in several ways, including changes in neuronal excitability, synaptic plasticity, and neural network activity. For instance, research has demonstrated that microgravity can alter the expression of genes involved in neuronal development, differentiation, and survival, leading to changes in neuronal morphology and function (Pani et al., 2013). Additionally, microgravity has been shown to affect the activity of neurotransmitters, such as dopamine and serotonin, which play critical roles in regulating mood, motivation, and cognitive function. One of the most significant effects of microgravity on the neural system is the alteration of the blood-brain barrier (BBB), a specialized structure that regulates the exchange of molecules between the bloodstream and the brain (Bennett et al., 2019). In microgravity, the BBB becomes more permeable, allowing toxins and other substances to penetrate the brain and potentially causing neuroinflammation and oxidative stress. This can lead to a range of cognitive and behavioral changes, including impaired memory, attention, and decision-making abilities (Mei et al., 2024).

### 3.1 Vestibular complications

In space, the vestibular system is subjected to a series of physiological changes that can disrupt its normal functioning. One of the primary changes is the shift of fluids towards the upper body, which can cause the vestibular apparatus to become less sensitive to changes in head position (Demir and Aydın, 2021). This phenomenon, known as "fluid shift," is caused by the lack of gravity, which allows fluids to redistribute throughout the body. As a result, the vestibular system becomes less responsive to changes in acceleration and deceleration, leading to impaired balance and spatial orientation (Tanaka et al., 2017). Another significant change occurs in the otolith organs, which are responsible for detecting linear acceleration and deceleration. In microgravity, the otolith organs become less sensitive to changes in acceleration, leading to a decline in the vestibular system's ability to detect changes in movement. This can cause astronauts to experience disorientation, vertigo, and difficulty maintaining posture (Hallgren et al., 2016).

This disruption leads to a mismatch between what the body expects to happen and what actually occurs, resulting in a myriad of vestibular complications. One of the most common and debilitating effects of microgravity on the vestibular system is disorientation. Without a clear sense of up and down, astronauts may experience difficulty navigating their surroundings, even in the relatively confined spaces of a spacecraft (Bronstein et al., 2020). Disorientation can lead to reduced performance, decreased reaction times, and increased risk of accidents. Nausea and vomiting are two of the most unpleasant consequences of vestibular complications in microgravity. As the body struggles to adapt to the unfamiliar environment, the conflicting signals from the vestibular system can trigger a severe response, leading to bouts of nausea and vomiting. This can lead to dehydration, electrolyte imbalances, and decreased performance, further exacerbating the challenges of space travel (Lackner, 2014).

### 3.2 Autonomic nervous system effects

Microgravity also affects the autonomic nervous system (ANS), which regulates various involuntary functions, such as heart rate, blood pressure, and body temperature. In space, the ANS is exposed to a unique set of stressors, including changes in temperature, humidity, and radiation, which can lead to alterations in heart rate variability, blood pressure, and other autonomic functions (Shen and Frishman, 2019). These changes can have significant implications for astronaut health and performance, particularly during long-duration space missions.

### 3.3 Perception of self-motion, proprioception, and multisensory integration

Microgravity profoundly alters the integration and reliability of sensory inputs essential for spatial orientation and motor control (Carriot et al., 2015). The absence of a gravitational vector renders the otolith organs of the vestibular system ineffective in providing accurate cues, significantly reducing the precision of vestibular input. Consequently, astronauts increasingly depend on visual information for self-motion perception (vection). Empirical evidence shows that in space, visually induced sensations of motion are more rapid, intense, and prolonged reflecting a reweighting of sensory inputs in favor of vision. Interestingly, while these changes modify perceptual experience, they do not markedly impair distance estimation when visual cues are present. On Earth, similar perceptual biases such as overestimation of travel distance when supine appear to share mechanistic similarities with those seen in space (Jörges et al., 2024).

Simultaneously, microgravity disrupts proprioceptive input derived from muscle spindles and joint receptors, which typically inform the central nervous system about limb position, posture, and body mass. This sensory distortion results in decreased proprioceptive accuracy, leading to misperceptions of body position and contributing to underpowered, slower voluntary movements. Astronauts adapt to this altered sensorimotor environment by increasing reliance on visual input; however, this compensation remains incomplete. As a result, spatial disorientation, variability in movement trajectories, and impaired motor planning are common (Proske and Weber, 2023).

These changes are underpinned by a shift in multisensory integration strategies. Under terrestrial gravity, the brain combines vestibular, proprioceptive, and visual cues according to their respective reliability to produce coherent perceptions of motion and orientation. In microgravity, the diminished reliability of vestibular and proprioceptive inputs, especially in floating or supine positions, leads to a recalibration of sensory weighting in favor of vision. This shift accounts for several spaceflight-induced phenomena, including heightened vection sensitivity, altered postural control, and instability in spatial orientation (Carriot et al., 2021).

### 3.4 Neural and glial responses to microgravity

Overall, the effects of microgravity on the human neural system and neuron activities are complex and multifaceted, involving changes in neuronal excitability, synaptic plasticity, and neural network activity. Additionally, astrocytes also respond to hypergravity with morphological changes and reduced reactivity, underscoring the importance of the cytoskeleton in glial cell function (Lichterfeld et al., 2022).

Neuromapping studies have shown changes in both gray and white brain matter following spaceflight, indicating alterations in brain structure under microgravity conditions. These changes also affect motor control and multitasking abilities, suggesting that microgravity influences how the brain processes and coordinates movements and tasks. Brain-DTI (Diffusion Tensor Imaging) investigations by the European Space Agency (ESA) have detected shifts in the connectivity of various brain regions and changes in cerebrospinal fluid volume during spaceflight (Rezaei et al., 2024). These observations hint at adaptations in neuronal connections and fluid dynamics within the brain, possibly related to neuroplasticity the brain's ability to reorganize itself by forming new neural connections under microgravity (Hupfeld et al., 2021). The Neuronix investigation involves using 3D cultures of neurons in microgravity to test gene therapy approaches for neurological diseases like Alzheimer's and Parkinson's. This research highlights the potential of microgravity environments for advancing treatments for neurological disorders, leveraging the unique capabilities of the space environment to grow complex cellular structures that mimic those found in humans (Marotta et al., 2024).

## 4 Oxidative stress mechanisms

Studies in simulated microgravity and isolation reveal changes in cytokine expression in the brain and blood, implicating mitochondrial reactive oxygen species (ROS) in immune dysregulation and highlighting compromised antioxidant defenses that worsen oxidative damage. Space radiation, particularly high-energy particles like protons and HZE ions, intensifies oxidative injury by generating ROS that damage DNA, proteins, and lipids, leading to genomic instability, neuroinflammation, and degenerative diseases in systems including the CNS and cardiovascular system (Jomova et al., 2023). This radiation-induced oxidative stress can persist long after exposure, causing ongoing cellular damage and raising the risk of cancer and organ degeneration. The combined influence of microgravity and radiation further amplifies oxidative stress, contributing to bone loss, immune suppression, and tissue damage across bone, muscle, neural, and cardiovascular systems (Zhang et al., 2025, Yatagai et al., 2019). Key indicators include increased lipid peroxidation, DNA damage, mitochondrial dysfunction, and elevated oxidative stress biomarkers like isoprostanes and 8-hydroxydeoxyguanosine. These effects threaten astronaut health by impairing immune and cognitive function, and have prompted research into countermeasures such as antioxidant-rich diets and targeted therapies. Understanding these synergistic effects is essential for protecting astronaut health during long-duration missions beyond low Earth orbit (Lee et al., 2025, Stein, 2002).

## 5 Key brain regions affected by microgravity and their changes

Microgravity induces complex and region-specific alterations in brain structure and function, significantly impacting areas critical for sensorimotor integration, spatial processing, and cognitive flexibility (Rezaei et al., 2024). The vestibular nucleus exhibits altered baseline activity and gene expression changes reflecting sensory reweighting due to the loss of gravity cues, with increased neural activity markers such as c-fos indicating vestibular plasticity during and after spaceflight (Pompeiano et al., 2002). The cerebellum undergoes rapid synaptic reorganization and dendritic morphology changes, including Purkinje cell cytoplasmic alterations and upward displacement within the skull, contributing to impaired motor control and coordination (Louis, 2016). Functional connectivity increases in the right angular gyrus, a hub for spatial processing and sensory mismatch detection, persist for months post-flight, while the bilateral insular cortex shows transient connectivity decreases followed by recovery, reflecting its role in multisensory integration for spatial orientation (Jillings et al., 2023). Frontal cortex gray matter volume decreases and functional changes likely signal stress and cognitive adaptation, whereas the cingulate cortex demonstrates region-specific activity fluctuations linked to cognitive flexibility and autonomic regulation (Luciana and Collins, 2022). Hippocampal alterations encompass changes in gene expression related to synaptic plasticity and metabolism, with initial impairments in synaptic function balanced by maintained or enhanced spatial cognition and working memory, alongside modifications in extracellular space and fluid drainage (Liang et al., 2023). The thalamus, a critical sensory relay, experiences persistent connectivity reductions affecting sensorimotor integration. More generally, microgravity causes brain shifts upward in the cranial cavity and cerebrospinal fluid redistributions that variably influence regional gray matter volumes, notably increasing sensorimotor cortex volume linked to reliance on somatosensory input (Jillings et al., 2023). Together, these neuroplastic and morphological adaptations within multisensory integration networks facilitate astronaut adjustment to the altered sensory and motor demands of space but may also underlie sensorimotor and cognitive challenges during and following missions (Tays et al., 2021). Understanding these nuanced brain region-specific changes is essential for developing effective countermeasures to preserve neural health in long-duration spaceflight (Roalf et al., 2025).

## 6 The nervous system disorders that may preclude space tourism

Current evidence highlights several nervous system disorders that could contraindicate space tourism due to the central nervous system (CNS) risks associated with spaceflight (Marfia et al., 2022). Spaceflight Associated Neuro-Ocular Syndrome (SANS), affecting approximately 70 % of long-duration astronauts, is characterized by optic disc edema, choroidal folds, optic nerve sheath distention, hyperopic shift, and posterior globe flattening, potentially causing irreversible vision loss and elevating the risk for terrestrial ocular and neurological disorders. Elevated intracranial pressure (ICP), likely resulting from cephalad fluid shifts in microgravity, can induce headaches, visual disturbances, and increased risk of hemorrhage or spinal cord injury; its chronic presence overlaps with SANS and may exacerbate vascular brain pathology (Kadipasaoglu et al., 2025). Neurodegenerative and neurocognitive impairments arise from space radiation and microgravity-induced oxidative stress, synaptic and dendritic injury especially in hippocampal and cortical regions neuroinflammation, and vascular dysfunction, collectively mirroring neurodegenerative diseases and causing lasting deficits in memory, cognition, and behavior (Miller et al., 2022). Vestibular and sensorimotor disorders, including acute space motion sickness, chronic vestibular dysfunction, and impaired coordination and balance, may persist, compromising mission safety and performance, particularly for individuals with pre-existing vestibular vulnerabilities (Clément et al., 2022). Lastly, psychological and psychiatric syndromes such as “space fog,” asthenia, fatigue, cognitive slowing, mood volatility, irritability, and sleep disturbances, although less clearly defined, have been documented and pose risks to cognitive function and decision-making during space travel (Marazziti et al., 2022). Collectively, these CNS disorders necessitate thorough screening and development of targeted countermeasures to ensure the safety of prospective space tourists (Sishc et al., 2022).

## 7 Radiation exposure and the neural system

### 7.1 Sources and nature of space radiation

As humans venture further into space, they are exposed to a unique set of challenges, one of which is radiation exposure. Radiation in space is a significant concern due to its potential effects on the human neural system. Radiation exposure in space can occur from various sources, including solar flares, galactic cosmic rays, and nuclear reactions in the Earth's atmosphere (Clément et al., 2020). These high-energy particles can penetrate the spacecraft and interact with the human body, causing damage to the neural system. The effects of radiation exposure on the neural system can be acute or chronic, depending on the dose and duration of exposure (Townsend, 2005).

### 7.2 Acute and chronic effects on the neural system

Acute effects of radiation exposure on the neural system include radiation sickness, which can manifest as nausea, vomiting, diarrhea, fatigue, and cognitive impairment. These symptoms can occur within hours or days of exposure and can be severe enough to impair the astronaut's ability to perform their duties (Blue et al., 2019). In severe cases, radiation exposure can cause seizures, coma, and even death. Chronic effects of radiation exposure on the neural system are more insidious and can occur months or years after exposure. These effects include cognitive impairment, memory loss, and emotional changes. Chronic radiation exposure can also increase the risk of neurodegenerative diseases, such as Alzheimer's and Parkinson's disease (Srivastava et al., 2023). More worrisome long-term effects of radiation include increased risk of cancer and irreversible structural brain damage. Developing strategies to either minimize or eliminate radiation exposure is particularly challenging when it comes to deep space travel. It will be necessary to protect future astronauts from this particular hazard (Dobney et al., 2023).

The risks associated with radiation exposure in space are significant, and NASA and other space agencies have implemented various measures to mitigate these risks. Another area of concern is the effects of radiation on the neural system. Space is filled with harmful radiation that can penetrate the brain and nervous system, causing damage to cells and tissues. Prolonged exposure to radiation can lead to cognitive impairment, memory loss, and even cancer. Radiation can also disrupt the normal functioning of the brain's neural networks, leading to changes in mood, behavior, and cognitive performance (Britten and Limoli, 2023).

Because of the absence of Earth's shielding magnetic field and the greater linear energy transfer (LET) of particles in deep space than in LEO, humans will be exposed to higher levels of ionizing radiation in deep space (Britten and Limoli, 2023). This will be particularly important for the Lunar Gateway program, which will be the next stage of human spaceflight and will include a lunar orbiting residence to support lunar surface operations.

Research using animal models has shown that exposure to artificial cosmic radiation leads to cognitive deficits in high-level learning and spatial memory (Miry et al., 2021). Some of this data also suggests the possibility of mental health problems, such as the appearance of persistent anxiety. The effects of the radiation environment on structural and functional brain systems have also been studied in rodent models (Zundel et al., 2022). These studies have suggested that exposure to radiation at spatially relevant fluence levels may cause extensive anatomical, cognitive, and behavioral changes (Kiffer et al., 2019).

Another approach to reducing radiation exposure is to use active shielding, which involves generating a magnetic field around the spacecraft to deflect charged particles. This technology is still in its infancy, but it has the potential to provide significant protection against radiation (Geng et al., 2015). Astronauts are also provided with personal protective equipment, such as space suits, to reduce radiation exposure during spacewalks. These suits are designed to provide a safe environment for astronauts while they are outside the spacecraft (Task Group on Radiation Protection in Space, ICRP Committee 2, 2013). One of the most promising areas of research is in the development of radioprotective drugs, which can be used to protect against radiation exposure. These drugs work by scavenging free radicals, which are molecules that can cause damage to cells and tissues (Checker et al., 2021). Radioprotective drugs have shown promise in reducing the effects of radiation exposure on the neural system, and they may play a critical role in protecting astronauts on long-duration space missions (Putt et al., 2022). Another area of research is in the development of advanced life support systems, which can recycle air, water, and waste, reducing the need for resupply missions and minimizing the risk of radiation exposure. These systems can also provide a safe environment for astronauts, reducing the risk of radiation exposure and other hazards (De Micco et al., 2023).

In addition to microgravity, space travelers are also exposed to radiation, which can have significant effects on the neural system. Radiation can cause damage to the brain and nervous system, leading to a range of effects, including: 1) Neuroinflammation: Radiation can cause inflammation in the brain, leading to damage to neural tissues and disruption of normal brain function. 2) Neurodegeneration: Prolonged exposure to radiation can cause neurodegeneration, leading to the loss of neurons and glial cells. 3) Cognitive Impairments: Radiation exposure can cause cognitive impairments, including decreased memory, attention, and problem-solving abilities (Wuyts et al., 2025, Jandial et al., 2018, Vazquez, 1998).

## 8 Risks of increased cancer incidence

Spaceflight increases overall cancer risk primarily due to exposure to space radiation, which includes high-energy protons and heavy ions (HZE particles) not encountered on Earth (Sridharan et al., 2015). These particles cause DNA damage, oxidative stress, and genomic instability, promoting cancer initiation, promotion, and progression through gene mutations, chromosomal aberrations, and disrupted cellular regulation. Space radiation is classified as a "complete carcinogen," capable of both initiating and promoting malignancies (Santibáñez-Andrade et al., 2023). Unlike terrestrial radiation, the high-energy, densely ionizing nature of space radiation induces more complex, clustered DNA damage, resulting in greater biological effectiveness and more aggressive tumors with higher metastatic potential (Mavragani et al., 2019). Animal studies and epidemiological data indicate astronauts face an increased incidence of various cancers, including brain cancer, underscoring the particular vulnerability of central nervous system tissue to radiation-induced damage during spaceflight (Parihar et al., 2015).

## 9 Countermeasures and mitigation strategies

To mitigate the effects of aerospace environments on the neural system, it is essential to develop countermeasures and mitigation strategies. Regular exercise and physical activity can help to mitigate the effects of microgravity on the neural system. Implementing regular sleep schedules can help to reduce sleep disturbances and fatigue (Stahn et al., 2023). Providing radiation protection, such as shielding and protective gear, can help to reduce the risks of radiation exposure. Cognitive training and mental stimulation can help to mitigate the effects of cognitive impairments. Providing psychological support and counseling can help to reduce the effects of stress and anxiety (Lagergren Lindberg et al., 2022).

Moreover, the study of the neural system in space has far-reaching implications for our understanding of the human brain and its function. By studying the effects of space travel on the neural system, we can gain insights into the neural mechanisms underlying cognition, emotion, and behavior, and develop new treatments for neurological and psychiatric disorders (Mann et al., 2019).

Despite these challenges, researchers are working to develop strategies to mitigate the effects of space travel on the neural system. One approach is to use neurofeedback training to help astronauts develop greater control over their brain function and improve their cognitive performance. Neurofeedback involves using sensors to monitor brain activity and providing real-time feedback to the individual, allowing them to learn how to self-regulate their brain function (Loriette et al., 2021).

Another approach is to use virtual reality and other forms of sensory stimulation to create a more engaging and stimulating environment for astronauts. This can help to reduce feelings of isolation and boredom, and provide a sense of connection to the outside world (Šlosar et al., 2023). In addition to these strategies, researchers are also working to develop new technologies to monitor and assess the neural system in real-time during space missions. This includes the use of portable neuroimaging devices, such as functional near-infrared spectroscopy (fNIRS), EEG and MEG which can provide real-time data on brain activity and function (Dinatolo and Cohen, 2022).

Research on isolation, such as the Mars500 study, has been very helpful in determining how isolation affects the central nervous system. A small group of volunteers was imprisoned in an environment designed to resemble a Mars mission. After an astonishing 520 days of isolation, researchers used diffusion tensor imaging to detect microstructural changes in the brain's white matter (Mumtaz et al., 2018). Neurocognitive deficits have been associated with microstructural changes and white matter injury in high-altitude pilot populations. Prolonged isolation in space may result in a variety of permanent changes, ranging from a decline in function in brain regions such as the sensorimotor network, where microstructural changes have been reported after launch (Doroshin et al., 2022).

On Earth, an EEG can detect abnormal brain activity caused by brain tumors, epilepsy, head injuries, and strokes (Attar, 2022). The NEUROSPAT experiment has demonstrated how this technology can be used on the International Space Station. It has demonstrated changes in brain activity, visuospatial function, visuo-attentional activity, and the effects of space travel on sleep. EEG has also been used in space to monitor changes in brain activity and neurocognitive function caused by isolation and microgravity (De la Torre, 2014). Typically, changes in activity are detected in the faster EEG frequencies (beta and alpha). Theta and alpha wave changes in simulated microgravity situations, particularly during bed rest, show suppression of cortical brain activity (Brauns et al., 2021). Reductions in sensorimotor input in simulated microgravity may account for many of the observed changes in EEG activity. Results also suggest that the loneliness and microgravity experienced during spaceflight lead to decreased sleep duration and slow-wave sleep (SWS). Reduced performance is another issue in microgravity (Barkaszi et al., 2022). Compared to terrestrial work, docking simulation tasks show an increase in global theta EEG oscillations during spaceflight. This has significant implications for astronauts as it has also been linked to poorer reaction times during the task (Zhang et al., 2023).

FMRI studies have shown that microgravity can alter the activity of brain regions involved in motor control, spatial awareness, and cognitive processing. MEG studies have revealed that microgravity can alter the neural oscillations underlying sensory processing, including the processing of visual and auditory information. In addition to these neuroimaging and neurophysiological techniques, researchers have also employed a range of behavioral and cognitive tasks to assess the effects of microgravity on astronaut performance and behavior. These tasks have included tests of attention, memory, spatial awareness, and decision-making, as well as measures of mood, motivation, and sleep quality (Sharma et al., 2025, Cassady et al., 2016).

Although data from these studies conducted outside of low Earth orbit are not currently available, they have provided important information on the effects of spaceflight on humans. In addition, much of the research is derived from pre- and post-flight measurements to determine the structural, functional, and behavioral effects of the mission (Hart, 2023). The drawback of pre- and post-flight studies is that many risk variables are typically exposed to participants at the same time. After leaving the space environment, researchers would be less likely to be biased or influenced by extraneous factors (e.g., changes related to orthostatic intolerance) thanks to in-flight monitoring technology that would allow them to measure changes in the CNS while in the space environment (Tocci et al., 2024).

## 10 Challenges and limitations

Astronauts face significant psychological challenges during long-duration space missions, including feelings of confinement, irritability, and tension among crew members due to the cramped living conditions of spacecraft. This confinement can lead to increased conflict and decreased morale, which may jeopardize mission success, particularly when teamwork is essential (Palinkas, 2001). Moreover, the monotony of life in space can contribute to decreased motivation and a phenomenon known as the "Groundhog Day effect," where daily routines feel repetitive and uninspiring. These psychological factors necessitate ongoing support and interventions to ensure the mental well-being of astronauts throughout their missions (Manzey, 2000). Research indicates that astronauts may experience cognitive impairments due to various factors, including isolation, stress, and microgravity conditions (Yin et al., 2023). For instance, the frequency of low scores on cognitive tests such as working memory (F2B), processing speed (DSST), and sustained attention (PVT) were notably higher than baseline levels prior to flight. In particular, 11.8 % of all individual test scores fell at or below 1.5 standard deviations of the baseline mean, with working memory tasks exhibiting an 18.7 % low score frequency. These findings suggest that the cognitive challenges posed by the space environment may require additional attention and research to address individual vulnerabilities in cognitive function (Dev et al., 2024, Basner et al., 2020).

## 11 Conclusion

The effects of aerospace environments on the neural system physiology are complex and multifaceted. Prolonged exposure to microgravity, radiation, and other environmental stressors can have significant effects on the nervous system, leading to changes in cognitive function, motor control, and overall well-being. To mitigate these effects, it is essential to develop countermeasures and mitigation strategies, including exercise, sleep schedules, radiation protection, cognitive training, and psychological support. By understanding the effects of aerospace environments on the neural system, we can work to ensure the health and well-being of space travelers and improve the success of space missions. As we continue to push the boundaries of space exploration, it is essential to continue researching the effects of aerospace environments on the neural system. Studying the long-term effects of space travel on the neural system, including the effects of prolonged exposure to microgravity and radiation. Investigating individual variability in response to aerospace environments, including genetic and environmental factors that influence susceptibility to neural system effects. The risks associated with radiation exposure are significant, but NASA and other space agencies are working to mitigate these risks through the use of shielding materials, active shielding, personal protective equipment, and research into radioprotective drugs and advanced life support systems. As humans venture further into space, it is essential to continue researching and developing new technologies to protect against radiation exposure and ensure the safety of astronauts on long-duration space missions. Investigating the neural system's ability to adapt to aerospace environments, including the role of neuroplasticity and neural compensation. By continuing to research the effects of aerospace environments on the neural system, we can work to ensure the health and well-being of space travelers and improve the success of space missions.

## Ethical approval

Not applicable

## Consent to participate

Not applicable.

## Consent for publication

Not applicable.

## Code availability

Not applicable.

## Clinical trial

Not applicable.

## Funding

No funding was received.

## CRediT authorship contribution statement

**Taherkhani Soroush:** Investigation, Funding acquisition, Formal analysis, Data curation, Conceptualization. **Fateme Arjmand:** Writing – review & editing, Writing – original draft. **Sohani Maryam:** Writing – review & editing. **Shahrezaei Aidin:** Writing – original draft, Data curation, Conceptualization. **Sepideh Marjaei:** Writing – review & editing. **Sanaz Sadeghi Esfahani:** Writing – review & editing. **Farinaz Nasirinezhad:** Writing – original draft, Methodology, Investigation, Funding acquisition, Formal analysis, Data curation, Conceptualization.

## Declaration of Competing Interest

The authors declare no competing interests that may have influenced in this manuscript.

## Data Availability

No new data was created or analyzed in this study.
